# Mutation-specific roles of sustained sodium current (I_Na_) in guiding precision medicine for long QT syndrome type 3

**DOI:** 10.1093/pnasnexus/pgaf379

**Published:** 2025-12-08

**Authors:** Vichaya Auvichayapat, Sarin Lekchuensakul, Pharawee Wandee, John Mauleekoonphairoj, Sirikorn Vongseenin, Praewphan Ingrungruanglert, Nipan Israsena, Phichaya Suthivanich, Damrongsak Jinarat, Apichai Khongphatthanayothin, Saknan Bongsebandhu-phubhakdi

**Affiliations:** Science Program in Physiology, Graduate School, Chulalongkorn University, 1873 Rama IV Rd., Pathum Wan, Bangkok 10330, Thailand; Department of Physiology, Faculty of Medicine, Chulalongkorn University, 1873 Rama IV Rd., Pathum Wan, Bangkok 10330, Thailand; Center of Excellence in Arrhythmia Research Chulalongkorn University, Department of Medicine, Faculty of Medicine, Chulalongkorn University, 1873 Rama IV Rd., Pathum Wan, Bangkok 10330, Thailand; Division of Cardiology, Department of Pediatrics, Faculty of Medicine, Chulalongkorn University, 1873 Rama IV Rd., Pathum Wan, Bangkok 10330, Thailand; Center of Excellence in Arrhythmia Research Chulalongkorn University, Department of Medicine, Faculty of Medicine, Chulalongkorn University, 1873 Rama IV Rd., Pathum Wan, Bangkok 10330, Thailand; Center of Excellence in Arrhythmia Research Chulalongkorn University, Department of Medicine, Faculty of Medicine, Chulalongkorn University, 1873 Rama IV Rd., Pathum Wan, Bangkok 10330, Thailand; Department of Physiology, Faculty of Medicine, Chulalongkorn University, 1873 Rama IV Rd., Pathum Wan, Bangkok 10330, Thailand; Center of Excellence for Stem Cell and Cell Therapy, Faculty of Medicine, Chulalongkorn University, 1873 Rama IV Rd., Pathum Wan, Bangkok 10330, Thailand; Excellence Center for Stem Cell and Cell Therapy, King Chulalongkorn Memorial Hospital, Thai Red Cross Society, 1873 Rama IV Rd., Pathum Wan, Bangkok 10330, Thailand; Center of Excellence for Stem Cell and Cell Therapy, Faculty of Medicine, Chulalongkorn University, 1873 Rama IV Rd., Pathum Wan, Bangkok 10330, Thailand; Excellence Center for Stem Cell and Cell Therapy, King Chulalongkorn Memorial Hospital, Thai Red Cross Society, 1873 Rama IV Rd., Pathum Wan, Bangkok 10330, Thailand; Science Program in Physiology, Graduate School, Chulalongkorn University, 1873 Rama IV Rd., Pathum Wan, Bangkok 10330, Thailand; Department of Physiology, Faculty of Medicine, Chulalongkorn University, 1873 Rama IV Rd., Pathum Wan, Bangkok 10330, Thailand; Center of Excellence in Arrhythmia Research Chulalongkorn University, Department of Medicine, Faculty of Medicine, Chulalongkorn University, 1873 Rama IV Rd., Pathum Wan, Bangkok 10330, Thailand; Cardiac Electrophysiology Research and Training Center, Faculty of Medicine, Chiang Mai University, 110 Inthawarorot Rd., Sri Phum, Muang, Chiang Mai 50200, Thailand; Center of Excellence in Arrhythmia Research Chulalongkorn University, Department of Medicine, Faculty of Medicine, Chulalongkorn University, 1873 Rama IV Rd., Pathum Wan, Bangkok 10330, Thailand; Division of Cardiology, Department of Pediatrics, Faculty of Medicine, Chulalongkorn University, 1873 Rama IV Rd., Pathum Wan, Bangkok 10330, Thailand; Department of Cardiology, Bangkok Hospital, 2 Soi Soonvijai 7, New Petchburi Road, Huaykwang, Bangkok 10310, Thailand; Department of Physiology, Faculty of Medicine, Chulalongkorn University, 1873 Rama IV Rd., Pathum Wan, Bangkok 10330, Thailand; Chula Neuroscience Center, King Chulalongkorn Memorial Hospital, The Thai Red Cross Society, 1873 Rama IV Rd., Pathum Wan, Bangkok 10330, Thailand

**Keywords:** antiarrhythmic drugs, molecular modeling, patch clamp recording, precision medicine, sustained sodium current

## Abstract

Long QT syndrome type 3 (LQTS3), caused by gain-of-function mutations in the *SCN5A* gene, encompasses a spectrum of clinical presentations, ranging from asymptomatic carriers to severe arrhythmic phenotypes, representing the “silent killer” and “brutal killer” dichotomy. The p.I239V mutation is associated with mild symptoms and minimal arrhythmic events, whereas the newly identified p.M1487K mutation is linked to life-threatening arrhythmic storms. This study aimed to explore the electrophysiological properties of these mutations and their responses to sodium channel blockers to advance precision medicine in LQTS3 management. Genetic analysis identified rare *SCN5A* mutations in two LQTS3 patients. Site-directed mutagenesis was used to construct mutant *SCN5A* plasmids, which were expressed in HEK293 cells. Electrophysiological properties were analyzed using patch-clamp techniques, and pharmacological responses to flecainide, mexiletine, and ranolazine were evaluated. Electrophysiological recordings correlated with clinical presentations. Both mutations showed increased window I_Na_ and faster recovery from inactivation. The p.I239V mutation lacked sustained I_Na_, while p.M1487K exhibited significantly increased sustained I_Na_ (2.3 ± 2.15%, *P* < 0.0001). Mexiletine and ranolazine effectively reduced sustained I_Na_ by 76.15 ± 5.83, and 77.63 ± 9.41%, respectively, outperforming flecainide, aligning with clinical responses. This study highlights the role of sustained I_Na_ in LQTS3 severity and underscores the importance of mutation-specific treatments. By tailoring treatments to the electrophysiological characteristics of each mutation, precision medicine offers a promising approach to improving patient outcomes in LQTS3.

Significance Statement Long QT syndrome type 3 (LQTS3) remains a leading cause of sudden cardiac death, yet patient outcomes vary dramatically depending on the underlying mutation. This study uncovers how distinct *SCN5A* mutations—the “silent killer” p.I239V and the “brutal killer” p.M1487K—shape electrophysiological abnormalities and dictate drug responsiveness. By linking sustained sodium current (I_Na_) to clinical severity and demonstrating the superior efficacy of mexiletine and ranolazine over conventional therapy, our work highlights sustained I_Na_ as a pivotal therapeutic target. These findings establish a framework for mutation-specific precision medicine, offering actionable strategies to improve survival in LQTS3 and setting a precedent for treating other inherited channelopathies.

## Introduction

Which one is scarier between a “silent killer” and a “brutal killer”? LQTS3 exemplifies this dilemma through its highly variable clinical presentations. Currently, there are several LQTS cases under the care of the Center of Excellence in Arrhythmia Research at Chulalongkorn University and the Department of Pediatrics at Chulalongkorn Hospital in Bangkok, Thailand. These patients present with distinct clinical severities, highlighting the challenge of variability in LQTS3. Two strikingly different cases of LQTS3 were selected to illustrate this variability. The first case is an 18-year-old female presenting with a milder phenotype, despite a family history of sudden cardiac death. She has experienced minimal symptoms and leads a relatively normal life, underscoring the potential for individuals with LQTS3 to remain undiagnosed, silently carrying the risk of sudden death. In contrast, the second case involves a 7-year-old female with a severe phenotype marked by recurrent arrhythmic storms, placing her at imminent risk of fatal arrhythmias. These cases exemplify the extremes of LQTS3, from the “silent killer” that allows the disease to persist undetected across generations to the “brutal killer” that demands immediate intervention.

LQTS3 is a life-threatening cardiac disorder caused by gain-of-function mutations in the *SCN5A* gene, which encodes the Na_V_1.5 sodium channel (1). Na_V_1.5 is a transmembrane protein that generates the inward sodium current (I_Na_), a critical component for initiating and propagating cardiac action potentials (2). Mutations in *SCN5A* lead to abnormal Na_V_1.5 function, disrupting the delicate balance of cardiac ion channel currents. This imbalance delays repolarization, prolonging the QT interval on the electrocardiogram (ECG), and increases the risk of fatal arrhythmias. LQTS3 is uniquely characterized by arrhythmias that occur predominantly at rest or during sleep, as opposed to other LQTS subtypes, where arrhythmias are often triggered by physical exertion or emotional stress (3–5). This predisposition to nocturnal events poses a silent yet persistent threat, emphasizing the importance of early recognition and management to prevent sudden death.

Genetic testing in the two cases revealed *SCN5A* mutations: c.715A>G (p.I239V) and c.4460T>A (p.M1487K), respectively. The I239V mutation was first identified in Finnish families with LQTS3 in 2004 (6) and has since reappeared in several studies; however, no electrophysiological characterization was conducted, leaving its role in LQTS3 unresolved (7–9). The M1487K mutation, reported here for the first time, is located within the IFM motif of the Na_V_1.5 inactivation gate—a highly conserved hydrophobic sequence in the intracellular III–IV linker essential for fast inactivation (10, 11). The precise functional effects of this mutation remain unknown. Furthermore, IFM motif mutations are exceedingly rare; to date, two clinical cases have been reported, F1468del (12) and F1486L (13), both exhibiting severe neonatal or infantile phenotypes characterized by markedly enhanced sustained I_Na_ (13, 14). Additional functional studies have demonstrated similar biophysical disruptions in the inactivation process even in the absence of identified carriers (15). These knowledge gaps hinder our understanding of the mechanistic basis for the clinical heterogeneity observed in LQTS3.

Diagnosing LQTS3 remains challenging due to its highly variable clinical presentations. While genetic testing is invaluable for identifying causative mutations, it serves primarily as a screening tool and does not always provide definitive predictions of clinical outcomes. Sustained I_Na_ is widely recognized as the primary mechanism driving LQTS3 pathophysiology (16). This persistent sodium influx prolongs the plateau phase of the cardiac action potential, predisposing to early afterdepolarizations and triggering arrhythmias (17). However, other mechanisms, including increased window I_Na_, faster recovery from inactivation, and altered steady-state activation, have also been implicated (3, 16). These mechanisms likely contribute to the diverse clinical manifestations observed in LQTS3, as exemplified by the stark contrast between the silent nature of the p.I239V mutation and the brutal outcomes of the p.M1487K mutation. Moreover, treatment remains challenging, as sodium channel blockers such as flecainide, mexiletine, and ranolazine have demonstrated variable efficacy, further complicating management strategies (18, 19).

This study aimed to address these challenges by evaluating the electrophysiological properties of the p.I239V and p.M1487K mutations to explore the mechanisms underlying their differing clinical severities. Furthermore, the study investigated the efficacy of sodium channel blockers on the p.M1487K mutation. By uncovering mutation-specific electrophysiological characteristics and treatment responses, this work seeks to enhance our understanding of LQTS3 pathophysiology and guide the development of targeted management strategies, ultimately contributing to the advancement of precision medicine for individuals with this condition.

## Results

### Clinical evaluation

**Fig. 1.**
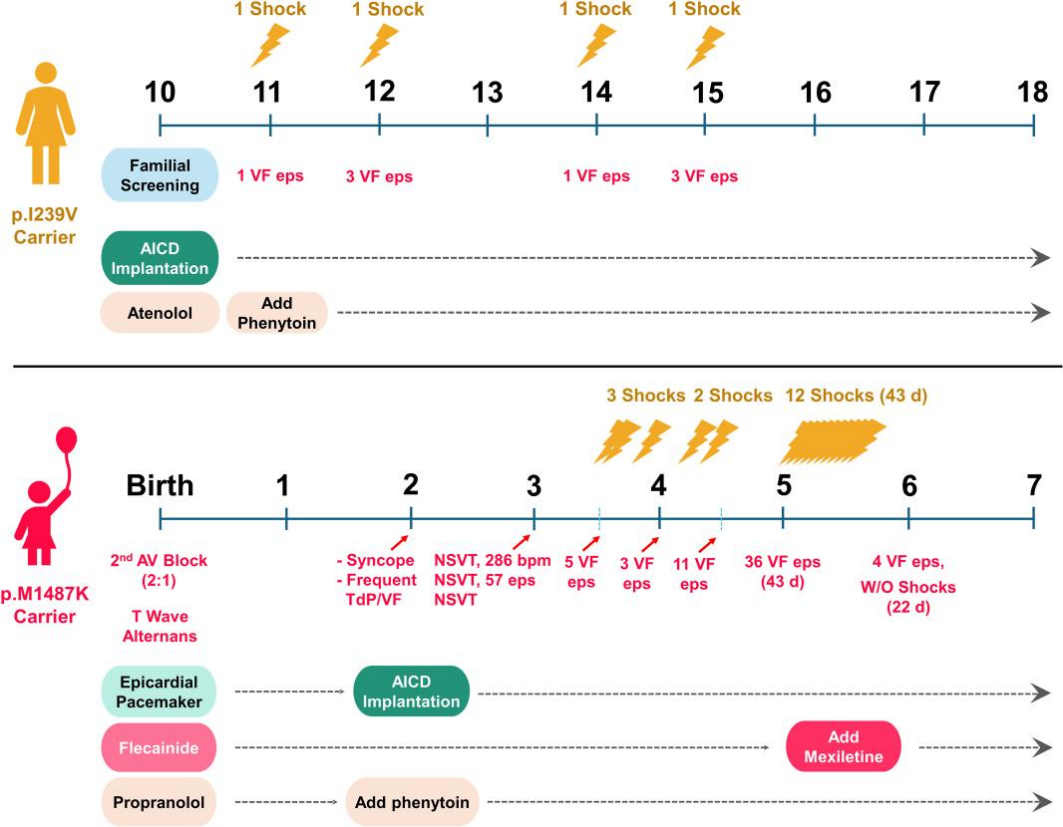
Timeline of clinical symptoms, treatments, and interventions in two patients with striking different clinical severity. The top panel represents a female adolescent patient diagnosed through familial screening at age 10. Ventricular fibrillation (VF) episodes began at age 11 and recurred periodically. She underwent AICD implantation and was initially treated with atenolol, with phenytoin later added to her regimen. The bottom panel illustrates the clinical course of a pediatric female patient diagnosed at birth with 2:1 atrioventricular (AV) block and T-wave alternans. The patient experienced frequent episodes of Torsades de Pointes (TdP) and VF, leading to AICD implantation at age 2. Despite initial treatment with flecainide and propranolol, recurrent VF episodes necessitated the addition of phenytoin. At age 5, the patient experienced multiple VF episodes, resulting in repeated ICD shocks, prompting the introduction of mexiletine, which subsequently reduced VF burden and ICD interventions. Lightning symbols indicate ICD shocks, and the number of VF episodes is annotated at the corresponding time points along the timeline. Treatment modifications are displayed as sequential additions of pharmacologic and device-based therapies.

**Fig. 2.**
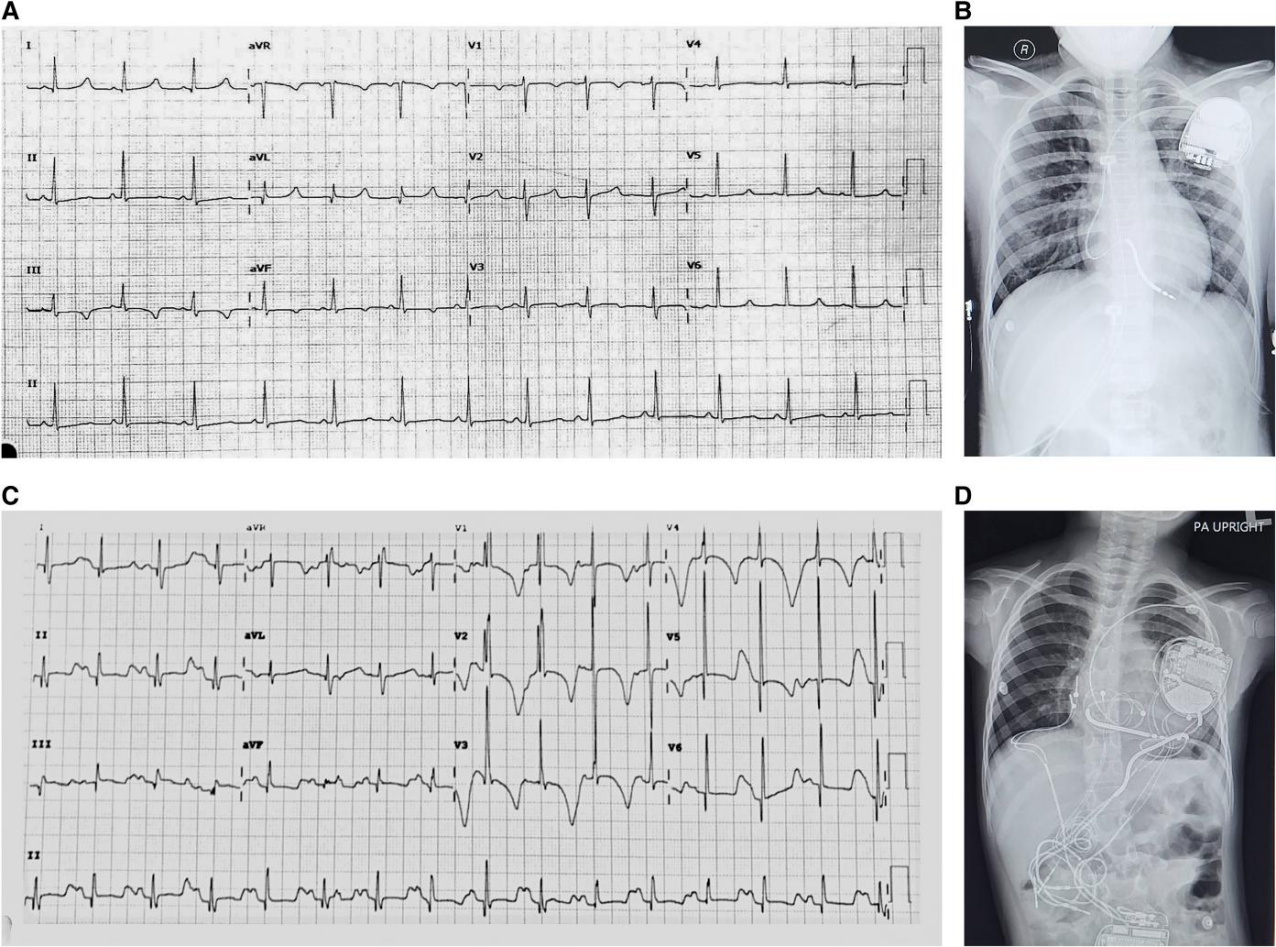
ECGs and AICD placement in two patients with markedly different clinical severity. A) ECG of the first patient, showing characteristic findings associated with their arrhythmic condition. B) Chest X-ray of the first patient after AICD implantation, demonstrating proper lead positioning. C) ECG of the second patient, revealing more severe electrophysiological abnormalities. D) Chest X-ray of the second patient after AICD implantation, showing multiple leads and a more complex device configuration.

Figure 1 illustrates the clinical course and management of two patients with *SCN5A* mutations leading to LQTS3. The upper panel depicts an adolescent female patient diagnosed at age 10 through familial screening. Initially, she was placed on atenolol and had an automatic implantable cardioverter-defibrillator (AICD) implanted. However, at age 11, she experienced her first ventricular fibrillation (VF) episode and received a shock, prompting the addition of phenytoin to her treatment regimen. Despite these interventions, she continued to experience recurrent VF episodes, with three episodes at age 12 and additional episodes at ages 14 and 15. Ongoing AICD therapy and antiarrhythmic medication provided long-term management beyond age 18. Figure 2A and B show the ECGs and chest X-ray of the first patient following AICD implantation, demonstrating proper lead positioning. Her QTc interval measured 543 ms.

The lower panel of Fig. 1 depicts a pediatric female patient who presented with severe arrhythmias from birth. She exhibited 2:1 atrioventricular (AV) block and T-wave alternans at birth, suggesting early conduction abnormalities, which led to the implantation of an epicardial pacemaker and initiation of flecainide and propranolol therapy. Despite this combined treatment, the arrhythmias persisted and progressively worsened. By age 2, the child experienced frequent torsades de pointes (TdP) and VF, along with recurrent syncope, leading to AICD implantation. At age 3, nonsustained ventricular tachycardia (NSVT) was documented on three occasions: first, at a rate of 286 bpm; second, with over 57 episodes recorded; and third, another NSVT episode. The arrhythmic burden worsened by age 3.5, with five VF episodes. At age 4, the patient experienced multiple VF episodes, receiving three AICD shocks. At age 5, a significant increase in arrhythmic events was noted, with 36 VF episodes over 43 days and 12 AICD shocks, underscoring the limited therapeutic efficacy of flecainide and propranolol in this severe LQT3 case. The addition of mexiletine led to a notable reduction in VF episodes, with only four events over 22 days and no further AICD shocks. Her ECG occasionally displayed T-wave alternans and a markedly prolonged QTc interval, peaking at 708 ms, indicating severe electrical instability and a high risk of life-threatening arrhythmia (Fig. 2C). The complexity of her device therapy is further demonstrated by a chest X-ray, revealing multiple pacing and defibrillation leads, reflecting her high arrhythmic burden and the need for advanced pacing strategies (Fig. 2D).

**Fig. 3.**
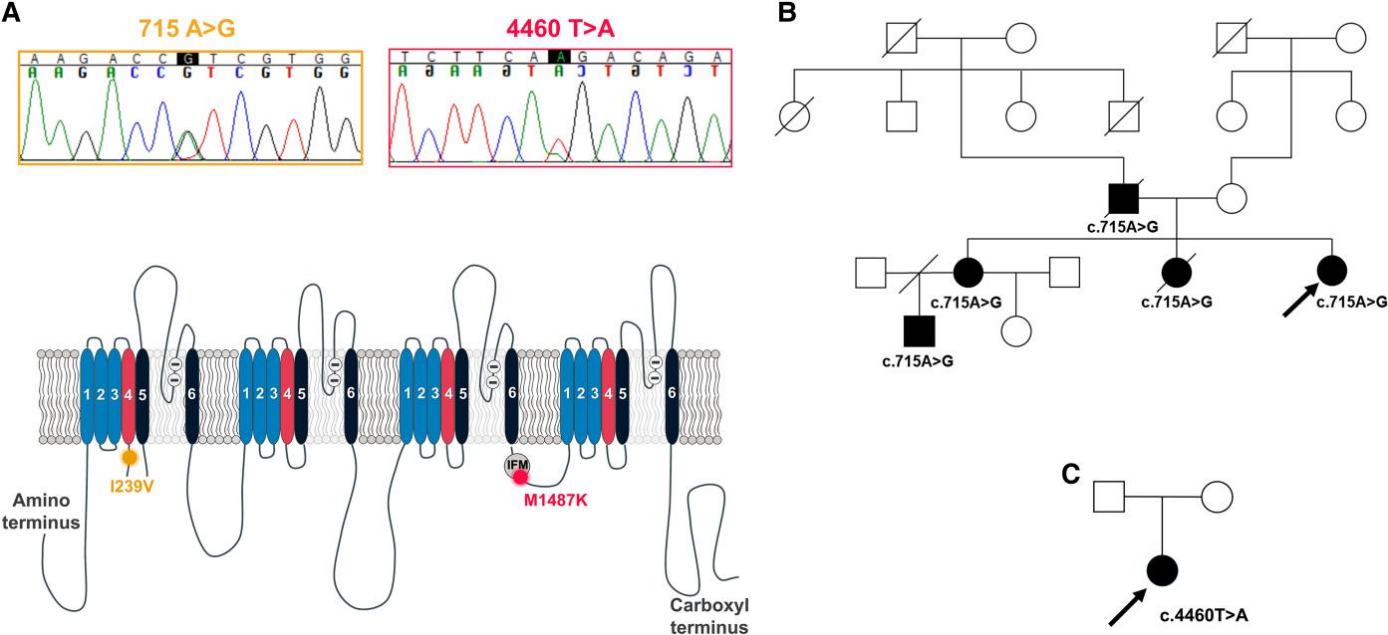
Genetic and familial analysis of *SCN5A* mutations in patients with LQTS3. A) Electropherograms displaying two *SCN5A* mutations: c.715A>G (resulting in the I239V substitution) and c.4460T>A (resulting in the M1487K substitution). Below, a schematic representation of the Na_V_1.5 highlights the locations of I239V in the DI-S4 segment and M1487K in the inactivation gate. B) Pedigree analysis of a family carrying the c.715A > G mutation. C) Pedigree of a patient with the de novo c.4460T > A mutation.

Sanger sequencing of the first patient and her family members revealed an *SCN5A* gene mutation at c.715A>G, leading to an amino acid substitution at position 239 in Na_V_1.5 (p.Ile239Val or I239V). This mutation occurs in the cytosolic S4-S5 linker of domain I, adjacent to the voltage sensor of this domain (Fig. 3A). The same mutation was identified in four other family members: her father, two older sisters, and the eldest sister's first son. Tragically, her father and one older sister had previously died from sudden cardiac death, as detailed in the pedigree (Fig. 3B). For the second case, Sanger sequencing revealed a mutation in *SCN5A* at c.4460T>A, resulting in an amino acid substitution at position 1487 in Na_V_1.5 (p.Met1487Lys or M1487K), which affects the methionine residue in the IFM motif of the inactivation gate (Fig. 3A). This mutation was not found in her parents, suggesting it is a de novo mutation (Fig. 3D).

These cases highlight the variability in disease progression associated with *SCN5A* mutations, underscoring the importance of individualized treatment. While the first patient had a relatively milder disease course with episodic VF events, the second patient exhibited severe, recurrent arrhythmias requiring multiple interventions. The findings emphasize the challenges in managing *SCN5A*-related arrhythmias and the critical role of mutation-specific therapies. Notably, the addition of mexiletine in the second patient significantly reduced the frequency of VF episodes, demonstrating the importance of personalized treatment strategies in severe cases.

### Electrophysiological properties

**Fig. 4.**
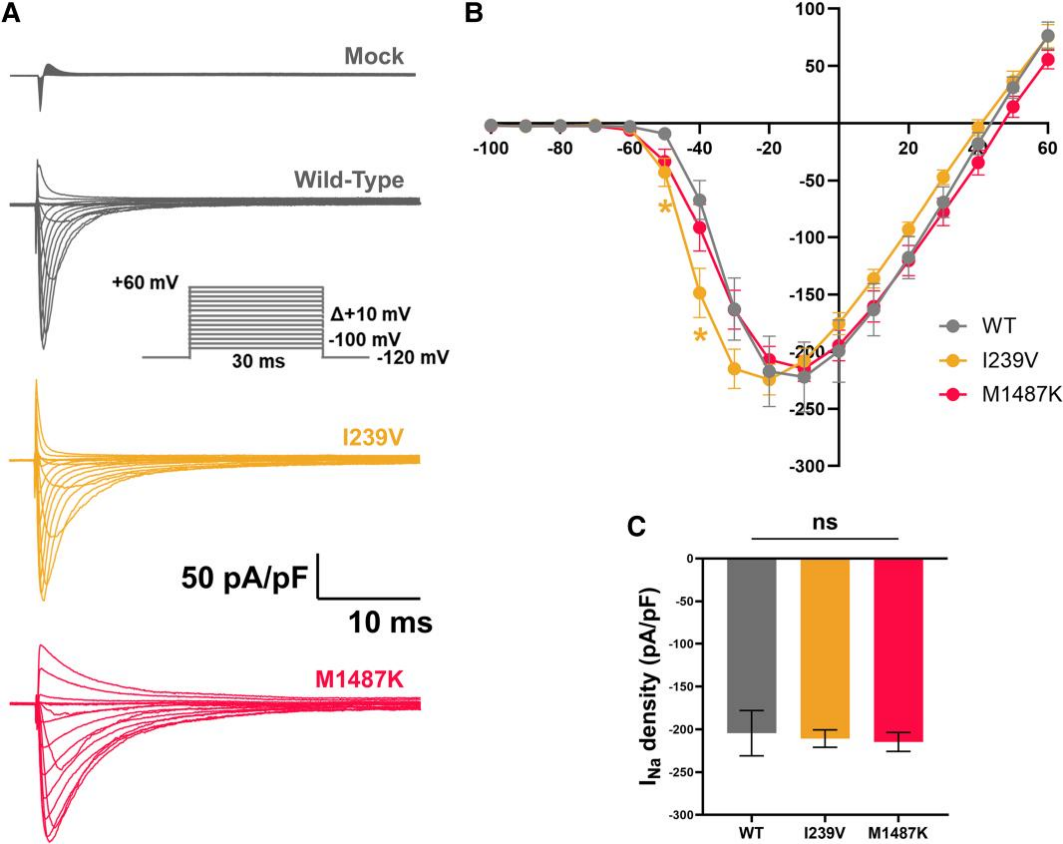
The comparison of peak I_Na_ of mock, WT, I239V, and M1487K. A) The representative of I_Na_ generated by the voltage step pulse protocol. B) The current–voltage curve plotted by voltage used and I_Na_ density generated from each voltage. C) Comparison of peak I_Na_ density at −10 mV. WT, and both mutant groups (*n* = 17 each), the data were presented in mean ± SEM. One-way ANOVA, and Tukey post hoc test. * P < 0.05, ns, no significant differences.

The results did not demonstrate I_Na_ produced by any voltage stimulations in the mock transfected group, contrasting with the WT Na_V_1.5 group as well as the p.I239V mutated group (I239V) and the p.M1487K mutated group (M1487K) (Fig. 4A). All electrophysiological parameters are summarized in Table 1.

**Table 1.** The values from electrophysiological assessment of I239V, and M1487K mutations on electrophysiological properties of Na_V_1.5.

	Na_V_1.5/WT	Na_V_1.5/I239V	Na_V_1.5/M1487K
1. Peak I_Na_ density	−204.5 ± 26.51 pA/pF	−210.9 ± 10.15 pA/pF^ns^	−214.8 ± 11.11 pA/pF^ns^
2. % Sustained I_Na_	0.02 ± 0.01%	0.34 ± 0.09%^ns^	2.3 ± 2.15%****
3. Steady-state of activation			
V_1/2_ of activation	−28.77 ± 1.55 mV	−36.64 ± 2.12 mV*	−32.22 ± 2.60 mV^ns^
K of activation	6.597 ± 0.39	6.22 ± 0.74^ns^	6.43 ± 0.4^ns^
4. Steady-state of inactivation			
V_1/2_ of inactivation	−82.16 ± 0.66 mV	−80.83 ± 2.04 mV^ns^	−78.17 ± 2.77 mV^ns^
K of inactivation	−5.40 ± 0.19	−5.87 ± 0.15^ns^	−5.58 ± 0.14^ns^
5. Window I_Na_ Probability (area under the curve)	0.02 ± 0.01	0.06 ± 0.02^ns^	0.11 ± 0.02*
6. Recovery from inactivation			
tau	4.15 ± 0.27 ms	4.09 ± 0.53 ms^ns^	4.15 ± 0.42 ms^ns^
7. Antiarrhythmic drugs effect			
Flecainide			40.52 ± 9.38 % (*n* = 8)
Mexiletine			76.15 ± 5.83 % (*n* = 8)
Ranolazine			77.63 ± 9.41 % (*n* = 8)

The data were presented in mean ± SEM, one-way ANOVA, and Tukey post hoc test. *P < 0.05, **P < 0.01, ***P < 0.001, ****P < 0.0001, ns, no significant difference.

### Peak I_Na_

According to the voltage step pulse protocol, the peak I_Na_ across various stimulation voltages ranging from −100 to 60 mV is shown in the I–V curve (Fig. 4B). At −10 mV stimulation, the peak I_Na_ did not significantly differ among the WT, I239V, and M1487K groups (*P* = 0.29). The peak I_Na_ density was −204.5 ± 26.51 pA/pF for the WT group, −210.9 ± 10.15 pA/pF for the I239V group, and −214.8 ± 11.11 pA/pF for the M1487K group (Fig. 4C). Notably, the I239V group displayed significantly larger peak I_Na_ compared to WT at −50 and −40 mV, indicating a hyperpolarizing shift in activation.

### Sustained I_Na_

**Fig. 5.**
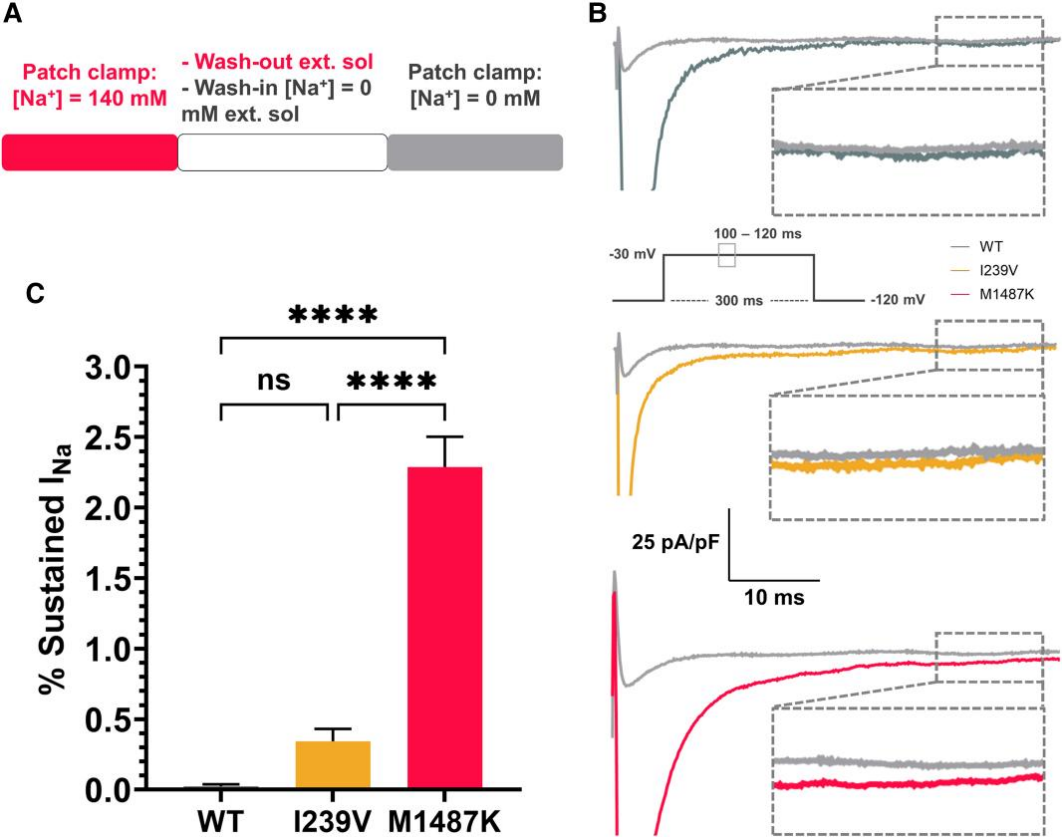
Comparison of sustained I_Na_ among WT, I239V, and M1487K. A) Voltage-clamp protocol used to evaluate sustained I_Na_. B) Representative I_Na_ traces for WT, I239V, and M1487K recorded before wash-out of external solution are shown. Traces recorded after wash-in of Na^+^-free external solution are also shown. Sustained I_Na_ is the remaining current after sodium removal. C) Percent sustained I_Na_, calculated as (sustained I_Na_/peak I_Na_ × 100). Data are presented as mean ± SEM. WT (*n* = 17), and both mutant groups (*n* = 30 each). Statistical analysis was performed using one-way ANOVA with Tukey's post hoc test. *P < 0.05, **P < 0.01, ***P < 0.001, ****P < 0.0001, ns, no significant differences.

To measure the sustained I_Na_, the external solution was washed out during patch clamp experiments, and then a sodium-free external solution was introduced (20) (Fig. 5A). Sustained I_Na_ was assessed at 100–120 ms after the peak I_Na_ and compared among WT, I239V, and M1487K groups. The data were expressed as the percentage of sustained I_Na_, (sustained I_Na_/peak I_Na_) × 100. Figure 5B illustrates the traces of sustained I_Na_ during −30 mV stimulation. In the WT group, no sustained I_Na_ was detected after the wash-out protocol. For the I239V group, most cells (two-thirds) also exhibited no sustained I_Na_, while a subset showed low levels of sustained I_Na_ ranging between 0.25 and 1%. Conversely, the M1487K group demonstrated significantly higher sustained I_Na_, with most cells exhibiting values above 1%. Quantitative analysis revealed that the sustained I_Na_ percentage for the I239V group was 0.34 ± 0.09%, which was not significantly different from the WT group (0.02 ± 0.01%, *P* = 0.36). In contrast, the M1487K group showed a significantly higher sustained I_Na_ percentage of 2.3 ± 2.15% compared to the WT group (*P* < 0.0001) (Fig. 5C).

### Steady-state of activation and inactivation

**Fig. 6.**
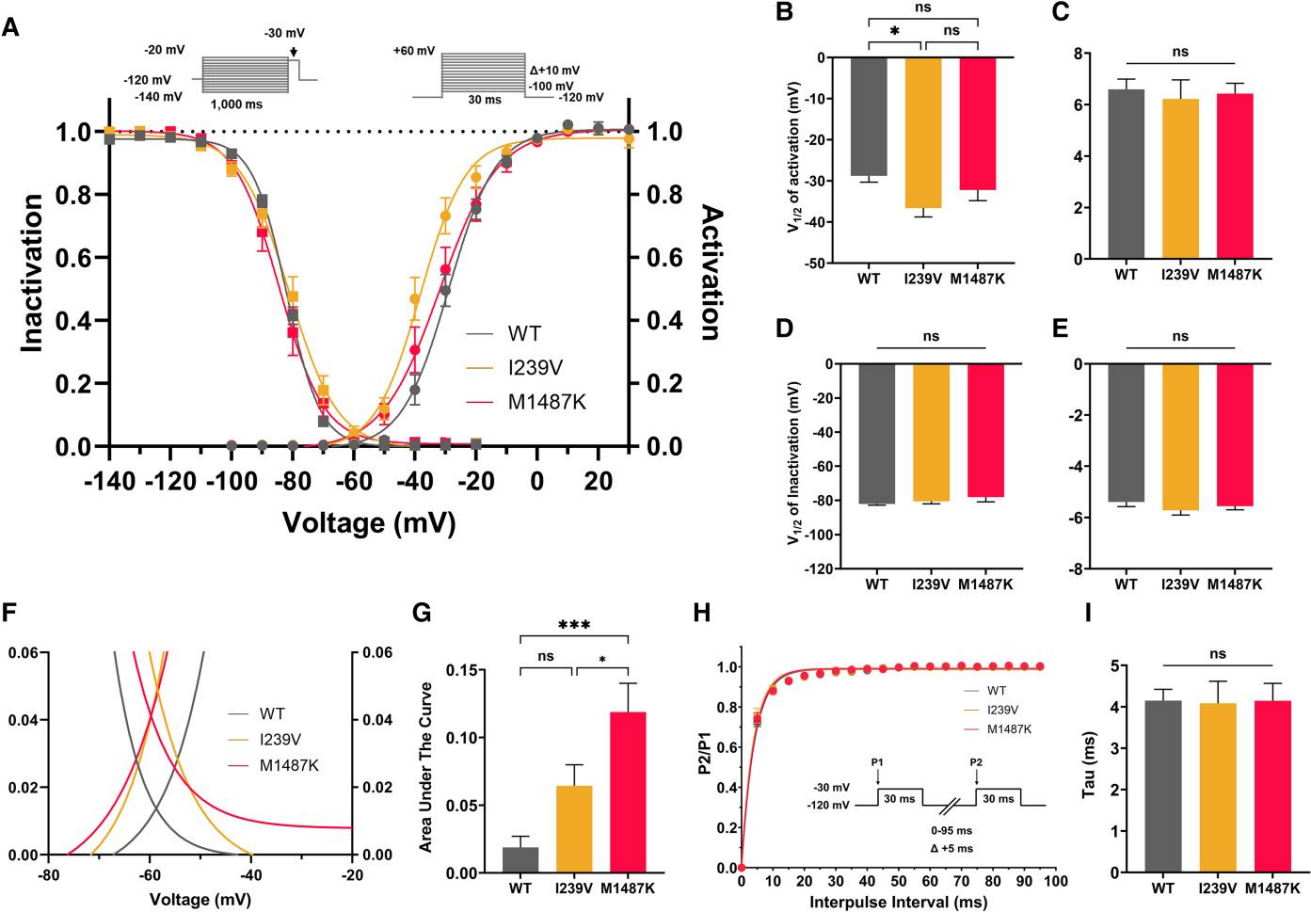
The comparison of steady-state of activation and inactivation curve, probability of window I_Na_, and recovery from inactivation. A) Steady-state activation and inactivation curves. The steady-state activation curve is represented by ● symbols, while the steady-state inactivation curve is denoted by ■ symbols. All curves were fitted using the Boltzmann equation. B) V_1/2_ of activation and C) slope factor (K) of activation. D) V_1/2_ of inactivation, E) K of inactivation, F) the probability of window I_Na_, G) the recovery from inactivation, H) the time constant (tau) of recovery from inactivation. The data were presented in mean ± SEM. WT (*n* = 17), and both mutant groups (*n* = 30 each). One-way ANOVA and Tukey post hoc test. * P < 0.05, ** *P* < 0.01, *** *P* < 0.001, **** *P* < 0.0001, ns, no significant differences.

The steady-state activation and inactivation curves were fitted using the Boltzmann equation (Eqs. S1 and S2). As shown in Fig. 6A, the I239V mutation produced a leftward (hyperpolarizing) shift in the activation curve compared to WT, whereas the M1487K group exhibited a modest but nonsignificant shift. The inactivation curves remained largely unchanged among the three groups.

Quantitative analysis confirmed these findings. The V_1/2_ of activation for WT was −28.77 ± 1.55 mV, whereas I239V showed a more negative shift at −36.64 ± 2.12 mV (*P* = 0.03). In contrast, M1487K displayed a V_1/2_ of −32.22 ± 2.60 mV, which was not significantly different from WT (*P* = 0.49) (Fig. 6B). The slope factor (k) of activation did not differ significantly among WT (6.60 ± 0.39), I239V (6.22 ± 0.74), and M1487K (6.43 ± 0.40) (*P* = 0.88) (Fig. 6C).

For steady-state inactivation, no significant differences were observed. The V_1/2_ values were −82.16 ± 0.66 mV (WT), −80.83 ± 2.04 mV (I239V), and −78.17 ± 2.77 mV (M1487K) (*P* = 0.08) (Fig. 6D). The slope factors were −5.40 ± 0.19, −5.87 ± 0.15, and −5.58 ± 0.14, respectively (*P* = 0.23) (Fig. 6E).

Interestingly, the steady-state inactivation curves revealed that WT and I239V channels underwent complete inactivation at high voltages, whereas M1487K channels retained a very small residual current, suggesting incomplete inactivation (Fig. 6F).

The probability of window I_Na_ was determined by plotting the overlap between the steady-state activation and inactivation curves using the following equation: (1/1 + exp((V_1/2_  _activation_ − V)/K_activation_)) × ((1 − C)/(1 + exp((V − V_1/2_  _inactivation_)/K_inactivation_)) + C. The analysis revealed that the area under the curve did not differ significantly between WT (0.02 ± 0.01) and I239V (0.06 ± 0.02) (*P* = 0.11). However, in the M1487K group, persistent availability of Na_V_1.5 channels at depolarized potentials led to a significantly larger window current area (0.11 ± 0.02), compared to WT (*P* = 0.0001) (Fig. 6G).

### Recovery from inactivation

The recovery from inactivation curves were fitted with a monoexponential function (Fig. 6H). The analysis showed no significant differences in recovery kinetics among the three groups (Fig. 6H). The time constant (τ), representing the time required for Na_V_1.5 channels to recover, was 4.15 ± 1.13 ms for WT, 4.09 ± 0.53 ms for I239V, and 4.15 ± 0.42 ms for M1487K (*P* = 0.99) (Fig. 6I). These results indicate that neither I239V nor M1487K significantly altered recovery from inactivation.

### Sodium channel blockers effect on p.M1487K mutation

**Fig. 7.**
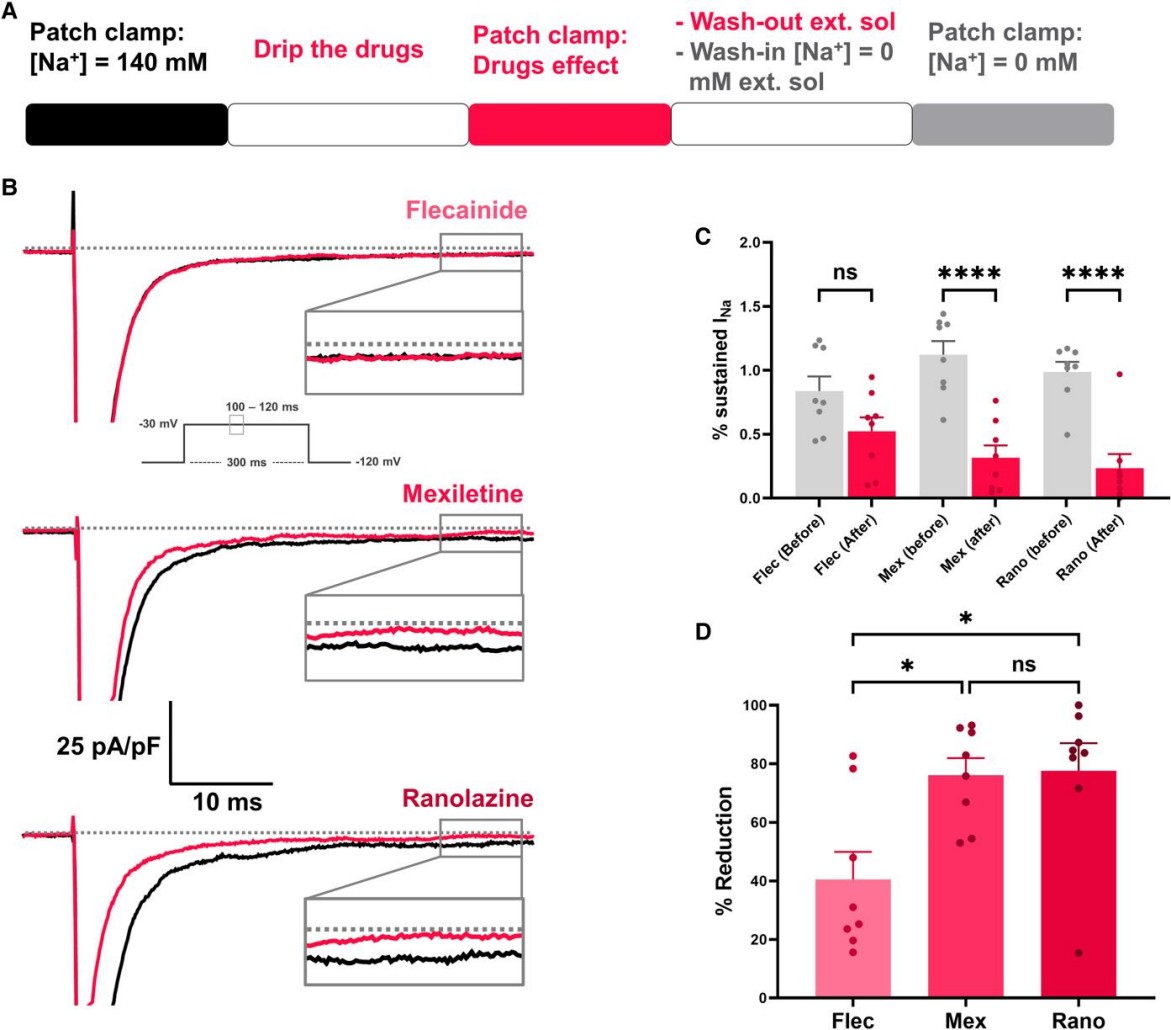
Effects of sodium channel blockers on the p.M1487K mutation. A) Experimental protocol used to assess the effects of sodium channel blockers on sustained I_Na_ reduction in the M1487K mutation. B) Representative current traces recorded before and after treatment with flecainide, mexiletine, and ranolazine following stimulation at −30 mV. C) Percentage of sustained I_Na_ before and after drug treatment. D) Comparison of sustained I_Na_ reduction among flecainide, mexiletine, and ranolazine. Data are presented as mean ± SEM. *n* = 8 per group. Statistical analysis was performed using one-way ANOVA followed by Tukey's post hoc test. *P < 0.05, **P < 0.01, ***P < 0.001, ****P < 0.0001; ns, not significant.

Since the sustained I_Na_ of the I239V mutation was minimal, only the efficacy of sodium channel blockers against the sustained I_Na_ of the M1487K mutation was analyzed. The efficacy of sodium channel blockers was assessed by comparing the sustained I_Na_ before and after the application of drugs, including flecainide, mexiletine, and ranolazine. Afterward, the external drug solution was washed out and replaced with an external solution without Na^+^ to establish the baseline for sustained I_Na_ (Fig. 7A). The trace of sustained I_Na_ was shown in Fig. 7B. The percentage of sustained I_Na_ before and after drug treatment was analyzed. Flecainide showed no significant reduction in sustained I_Na_ (*P* = 0.27), while both mexiletine and ranolazine significantly decreased sustained I_Na_ (*P* < 0.0001) (Fig. 7C).

The percentage reduction was calculated as [(The percentage of sustained I_Na_ before treatment—the percentage of sustained I_Na_ after treatment)/The percentage of sustained I_Na_ before treatment] × 100. The results showed that both mexiletine and ranolazine had similar effects, reducing the sustained I_Na_ by 76.15 ± 5.83, and 77.63 ± 9.41 of the maximum sustained I_Na_, respectively. In contrast, flecainide reduced the sustained I_Na_ by 40.52 ± 9.38% (Fig. 7D). These findings indicate that mexiletine and ranolazine are highly effective in suppressing sustained I_Na_, whereas flecainide has a limited effect.

### Molecular modeling

**Fig. 8.**
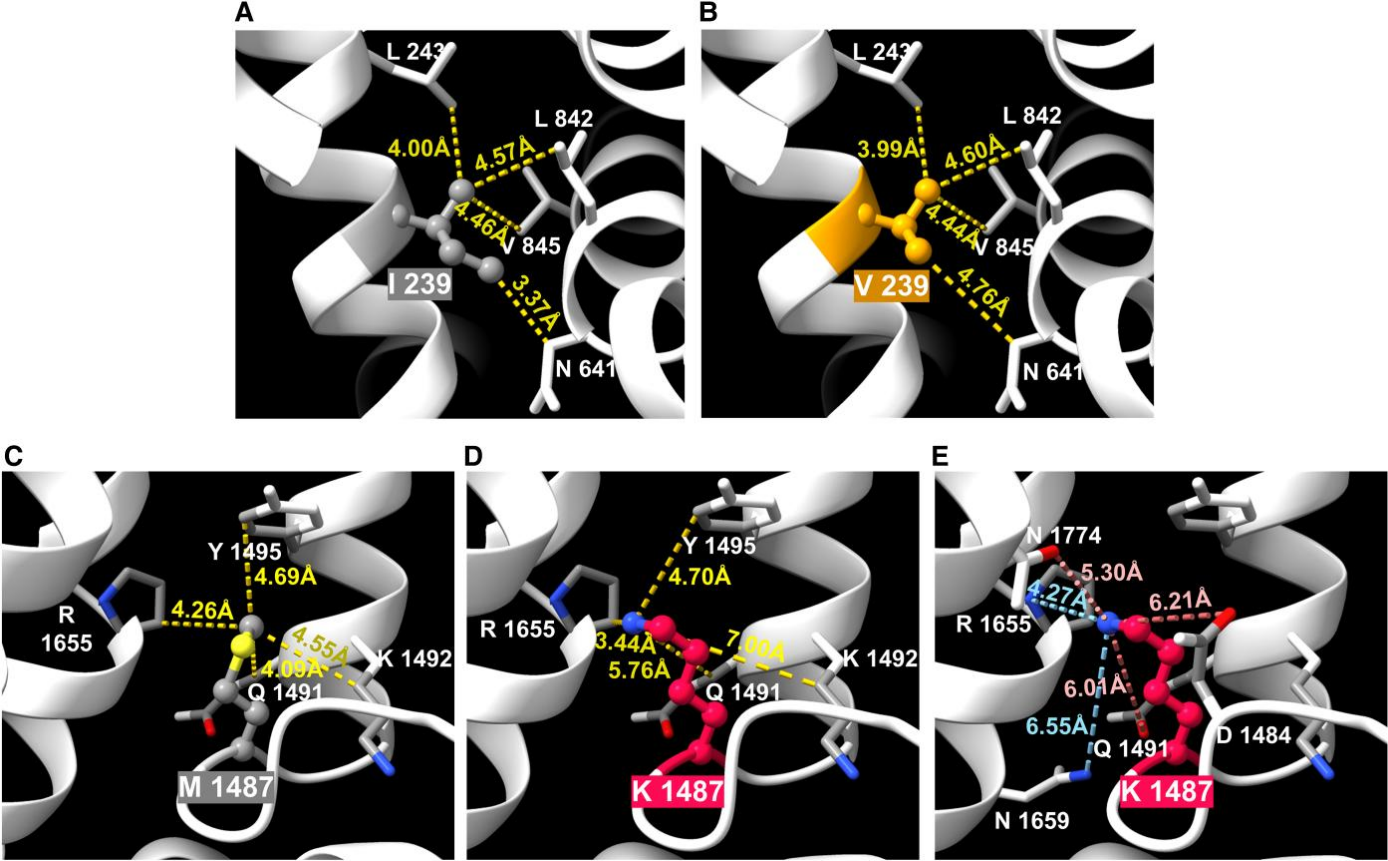
The molecular modeling and the distance to other close amino acid of mutated Na_V_1.5. A) WT isoleucine at position 239, B) valine of I239V mutation, C) WT methionine at position 1487, D) lysine of M1487K mutation, E) distances between lysine in the M1487K variant and nearby charged residues (positively and negatively charged residues are labeled).

Figure 8A and B showed the structural comparison between the WT isoleucine at position 239 (I239) and the mutated valine (V239) in the Na_V_1.5. The yellow dashed lines indicated the distances between the methyl group side chain of residue 239 and neighboring residues (L243, L842, V845, and N641) in the protein structure. The shift in distances between the methyl group of position 239 and its neighboring residues suggests a minor conformational adjustment in the protein structure due to the mutation. Although the distances do not change drastically, the mutation from a bulkier isoleucine to a smaller valine likely leads to a slightly altered packing of the protein, especially seen in the interaction with N641, where the distance increases from 3.37 Å to 4.76 Å.

The M1487K mutation occurs in the IFM motif, where methionine at position 1487 is replaced by lysine. While methionine and lysine have similar molecular sizes, the mutation causes the NH_3_ group of lysine to orient toward R1655, shortening the distance between them from 4.26Å to 3.44Å. The distance to K1492 increases significantly from 4.09Å to 7.00Å, while the distance to Y1495 remains relatively unchanged, shifting from 4.69Å to 4.70Å. However, the distance to Q1491 increases from 4.25Å to 5.76Å (Fig. 8C and D). Interestingly, Methionine is a nonpolar, hydrophobic amino acid with a thioether side chain, contributing to hydrophobic interactions and stability within the protein. In contrast, lysine is a positively charged, hydrophilic amino acid with a long side chain ending in an amino group, introducing a positive charge and the potential for new ionic interactions. The replacement of methionine with lysine significantly alters the local environment, as the amino group in lysine attracts negatively charged amino acids like N1774 (5.30Å), D1484 (6.21Å), and Q1491 (6.55Å), while repelling positively charged residues like R1655 (2.74Å) and N1659 (6.04Å) (Fig. 8E).

## Discussion

This study highlights the distinct clinical severities associated with the I239V and M1487K mutations in Na_V_1.5 and links these differences to underlying electrophysiological and structural alterations. The I239V carrier exhibited a relatively stable arrhythmic profile, with VF episodes beginning in adolescence, managed by AICD implantation and pharmacologic therapy. Although ICD shocks were required, their frequency was limited, reflecting the mutation's modest increase in sustained I_Na_ and nonsignificant elevation in window I_Na_. In contrast, the M1487K carrier presented with malignant arrhythmias from infancy, marked by early AV block, frequent TdP/VF, and a rapid escalation to multiple ICD shocks despite antiarrhythmic therapy. This severe phenotype corresponds to a pronounced increase in sustained I_Na_ and a significantly elevated window I_Na_ probability, underscoring the mutation's high proarrhythmic potential.

The I239V mutation is located between segments 4 and 5 of domain I, near Na_V_1.5's voltage sensor. It induces subtle structural changes, as molecular modeling reveals that replacing isoleucine with valine at position 239 reduces side-chain bulk, increasing the distance between residues I239 and V845. This enhances local flexibility near the voltage sensor, facilitating its movement during depolarization and resulting in a negative shift in activation gating. Despite these alterations, inactivation remains unaffected, correlating with a milder clinical phenotype. Similar patterns are observed in mutations like E1295K, where a carrier exhibited no significant symptoms despite a QTc interval of 480 ms (21). Another variant, A1330T, is associated with a sudden death at rest in one carrier, while her mother and her siblings who carried this variant remained asymptomatic, supporting the idea that the negative shift of steady-state of activation alone may not cause severe symptoms (22).

In contrast, the M1487K mutation is located at the IFM motif, where the substitution introduces a larger lysine side chain. However, side-chain size alone is insufficient to explain the increased sustained I_Na_. Instead, the lysine substitution introduces a positive charge, disrupting hydrophobic interactions, and forming new electrostatic and hydrogen bonds, destabilizing the local structure. This charge may repel nearby positively charged residues and attract negatively charged ones, altering protein conformation. Additionally, lysine's hydrophilic nature disrupts the local hydrophobic environment, destabilizing the inactivation gate, while its bulky side chain may interfere with the tight packing necessary for proper gate function, delaying channel closure after opening. These molecular changes align with the electrophysiological impacts of this mutation, as it significantly increases sustained I_Na_ and is associated with severe symptoms, including a QTc of 708 ms, second-degree AV block, or Torsades de Pointes.

Growing evidence suggests that increased sustained I_Na_ is a key determinant of clinical severity in LQTS3. To date, only two reported LQTS3 cases involve mutations at the highly conserved IFM motif—F1486del and F1486L. The F1486del mutation, identified as a de novo variant, was found in an infant with extreme clinical presentation, including a QTc of 860 ms, 2:1 atrioventricular block, and polymorphic ventricular tachycardia (12). Electrophysiological analysis revealed significantly reduced peak I_Na_, markedly enhanced sustained I_Na_, and impaired inactivation (14). Similarly, the F1486L mutation, identified in a sudden infant death syndrome (SIDS) case, also demonstrated a pronounced increase in sustained I_Na_ (13). Including the mutation investigated in this study, all three IFM motif mutations share a common mechanism: impaired inactivation leading to enhanced sustained I_Na_. Notably, all were de novo and associated with sudden death during infancy, underscoring this mechanism as among the most lethal in LQTS3. This is further supported by other mutations outside the IFM motif, such as F1473C and M1652R, which also show increased sustained I_Na_ and correlate with severe clinical manifestations, including extreme QT prolongation, atrioventricular block, and recurrent arrhythmias (23, 24).

While other electrophysiological abnormalities—such as faster recovery from inactivation—have been implicated in drug-induced LQTS, their clinical impact is generally more context-dependent. For example, *SCN5A* mutations like G615E enhance sodium channel availability during the action potential by accelerating recovery from inactivation (25–27). Under normal conditions, this allows more channels to reopen during late repolarization, modestly increasing window or sustained I_Na_ (28 , 29). In the presence of external stressors such as I_Kr_-blocking drugs, this mechanism can promote afterdepolarizations and Torsades de Pointes (25, 26). However, unlike sustained I_Na_, which directly prolongs repolarization and drives arrhythmia even in the absence of external triggers, faster recovery from inactivation alone is insufficient to explain the most severe phenotypes seen in LQTS3. Taken together, these observations highlight enhanced sustained I_Na_ as the primary electrophysiological abnormality associated with early-onset lethality and offer a compelling rationale for prioritizing it in risk stratification and therapeutic development.

From all the previous articles mentioned, it is suggested that sustained I_Na_ is the critical determinant of clinical severity. Mutations that do not cause sustained I_Na_ tend to act as “silent killers,” producing no immediate dangerous effects, allowing carriers to live and reproduce normally. However, this silent nature results in a larger population of carriers who remain at risk for sudden cardiac death. On the other hand, mutations that cause a significant increase in sustained I_Na_ are considered “brutal killers,” associated with severe phenotypes and high clinical severity. These mutations often lead to life-threatening symptoms early on, reducing survival and reproductive fitness, which results in a smaller number of carriers.

The sodium channel blocker effects observed in this study align with the patient's clinical response. The p.M1487K mutation was associated with poor response to flecainide but better efficacy with mexiletine. Flecainide, a class IC antiarrhythmic, primarily reduces peak I_Na_ and prolongs refractoriness but has limited ability to inhibit sustained I_Na_, explaining its reduced effectiveness (30). In contrast, mexiletine, a class IB sodium channel blocker, preferentially binds to two distinct sites on Na_V_1.5, with a preference for the inactivated state (31, 32). Mutations like p.M1487K that stabilize this state may enhance drug binding, improving clinical outcomes. Similarly, ranolazine, an antianginal drug, inhibits sustained I_Na_ by stabilizing Na_V_1.5 in its inactivated state (33, 34). Since sustained I_Na_ in p.M1487K results from defective inactivation of Na_V_1.5, drugs that effectively suppress this current, like mexiletine and ranolazine, offer superior therapeutic benefits. Although mexiletine had to be imported to Thailand, ranolazine is locally available, and both reduced sustained I_Na_ by ∼80%, making them more effective than flecainide for managing mutations like p.M1487K. This difference in drug efficacy likely stems from the specific mechanisms of mexiletine and ranolazine, which target and reduce sustained I_Na_, preventing prolonged depolarization and reducing arrhythmogenic risks. Ranolazine could thus serve as an alternative treatment option for this patient.

Several studies have evaluated the effectiveness of sodium channel blockers through in vitro testing followed by clinical application. For example, the ΔKPQ mutation, which significantly increases sustained I_Na_, was associated with prolonged QTc, where ranolazine was shown to shorten the prolonged QTc and improve diastolic relaxation in patients with the LQT3-ΔKPQ mutation (35, 36). Similarly, our study highlights the strong correlation between the effects of sodium channel blocker treatments observed in vitro and clinical outcomes in patients, suggesting that in vitro functional studies can reliably predict the therapeutic efficacy of drugs in vivo. This experiment also underscores the critical role of sustained I_Na_ in determining clinical severity, as its elevation is associated with an increased risk of arrhythmic events. The significant reduction of sustained I_Na_ post-treatment demonstrates its relevance as a therapeutic target, reinforcing the importance of mutation-specific approaches in managing LQTS3. This supports the potential for precision medicine approaches, tailored to the specific electrophysiological and molecular characteristics of individual mutations.

In conclusion, our study highlights the role of sustained I_Na_ in LQTS3 variability. Both the I239V and M1487K mutations enhance window I_Na_, but the M1487K mutation, the “Brutal Killer,” notably enhances sustained I_Na_, contributing to severe clinical phenotypes. The I239V mutation, a “Silent Killer,” presents subclinical arrhythmogenic risk. Conventional therapies like flecainide are ineffective for M1487K, but Mexiletine and Ranolazine offer more effective treatment. This study underscores the need for genetic testing and electrophysiological assessments to guide precision medicine and tailor therapies to optimize patient outcomes.

A limitation of this study is its focus on screening sustained I_Na_ in relation to clinical severity. A meta-analysis of previous studies linking sustained I_Na_ to clinical symptoms across various mutations would provide a broader understanding. Additionally, studies using advanced models, like iPSC-derived cardiomyocytes or animal models, could help validate these findings and assess the therapeutic potential of sodium channel blockers.

## Materials and methods

### Patient samples

This study was approved by the Institutional Review Board (IRB) of the Faculty of Medicine, Chulalongkorn University (IRB No. 0208/56). Clinical information was obtained from patients’ inpatient and outpatient medical records. The whole exome sequencing (Macrogen, Inc., Korea), including analysis of LQTS-causative genes (*KCNQ1*, *KCNH2*, *SCN5A*, *ANK2*, *KCNE1*, *KCNE2*, *KCNJ2*, *CACNA1C*, *CAV3*, *SCN4B*, *AKAP9*, *SNTA1*, *KCNJ5*, *CALM1*, *CALM2*, *CALM3*, and *TRDN*), was performed on the patient after obtaining informed consent. Mutation confirmation was subsequently conducted through Sanger sequencing (1st BASE, Malaysia) in the patient and their family.

### Cell culture

Human Embryonic Kidney 293 FT cells were kindly provided by Center for Excellence in Molecular Genetics of Cancer and Human Diseases, Chulalongkorn University and were cultured with Dulbecco's modified Eagle's medium (DMEM) supplemented with 10% heat-inactivated fetal bovine serum, 1% Penicillin–Streptomycin in a 5% CO_2_ incubator at 37 °C.

### *SCN5A* site-directed mutagenesis

The plasmid pCGI-*GFP-SCN5A* was provided by Prof. Connie Bezzina from Amsterdam University Medical Centre, University of Amsterdam, Netherlands. For constructing the c.715A>G (p.I239V) *SCN5A* plasmid, the QuikChange XL site-directed mutagenesis kit was used. For constructing the c.4460T>A (p.M1487K) *SCN5A* plasmid, the Phusion Site-Directed Mutagenesis Kit was used. The primers used for site-directed mutagenesis were provided in Table S1. Finally, the plasmid DNA was extracted using Exprep Plasmid SV and its sequences were confirmed by Fast next-generation sequencing (FastNGS; U2Bio, Thailand). In summary, three types of *SCN5A* plasmids were used in this experiment: *SCN5A* wild type (WT), *SCN5A*-I239V (I239V), and *SCN5A*-M1487K (M1487K), Additionally, mock plasmid, *pIRES2-GFP* (no *SCN5A*, serving as a negative control) were also used in this study.

### Transfection of HEK293

Two hundred and ninety three FT cells were transiently transfected with 2,500 ng of *pIRES2-GFP* or *pCGI-GFP-SCN5A*, including WT, I239V, or M1487K mutations, using Lipofectamine 3000 according to the company's protocol. Initially, cells were seeded to achieve 70–90% confluence on a 6-well plate. The Lipofectamine 3000 reagent was diluted with Opti-MEM Medium. Subsequently, the diluted DNA was added to each tube of diluted Lipofectamine 3000 Reagent at a 1:1 ratio and incubated for 20 min. Finally, the plasmid-lipid complex was added to the cells. Twenty-four hours after transfection, cells were reseeded onto 35-mm dishes, and patch-clamp recordings were performed on GFP-positive cells.

### Electrophysiological and Na^+^ gating properties acquisition and analysis

The intracellular solution was prepared with the following concentrations (in mM): 35 NaCl, 105 CsF, 10 EGTA, and 10 HEPES, pH 7.4 titrated with CsOH; osmolality 290 mOsm/kg. The extracellular (bath) solution was prepared with the following concentration: 140 NaCl, 5 KCl, 1 MgCl_2_, 2 CaCl_2_, 10 glucose, and 10 HEPES, pH 7.4 titrated with CsOH; osmolality 320 mOsm/kg. Microelectrodes (borosilicate glass capillaries BF150-86-10, Sutter Instrument, Novato, CA, USA) with exhibited resistances of 2–3 MΩ were used. The standard whole-cell patch-clamp technique was employed to measure mock transfect (negative control; no *SCN5A*), *SCN5A* WT, I239V, and M1487K I_Na_. This experiment was conducted at room temperature (25 °C) utilizing the AxoPatch 200B amplifier, Digidata 1440A, and pCLAMP software version 11.

### Drug treatment

Drug stock solutions were prepared with mexiletine and ranolazine in water, and flecainide in ethanol. To study their inhibitory effects on sustained I_Na_, the drugs were applied during HEK293FT patch-clamp experiments. The final concentrations used in this study were 10 μM for flecainide, and 50 μM for both mexiletine and ranolazine, following the protocol described by Bankston et al. (23).

### Molecular modeling

To determine the putative structural consequences of each mutation, molecular modeling was performed using the cryo-EM structure of human Na_V_1.5 from Protein Data Bank in Europe model PDB-CPX-172023 (37) and the protein structure homology modeling by Chimera X version 1.9.

### Quantification and statistical analysis

The data are expressed as mean ± SEM. The normality and homogeneity of variance were evaluated using the Shapiro–Wilk test and Levene's test, respectively. For data meeting the assumptions of normality and homogeneity, statistical comparisons were performed using one-way ANOVA followed by Tukey's post hoc test. If these assumptions were not met, the Kruskal–Wallis test was applied, followed by Dunn's pairwise test for multiple comparisons. All statistical analyses were conducted using SPSS version 28, and graphs were created using GraphPad Prism version 10.

## Supplementary Material

pgaf379_Supplementary_Data

## Data Availability

All data are available in the main text, figures, supplementary materials, and dataset.
