# Supplementary material for: Mutation-specific roles of sustained sodium current (INa) in guiding precision medicine for long QT syndrome type 3
Source: PNAS Nexus. 2025 Dec 8;4(12):pgaf379. doi: 10.1093/pnasnexus/pgaf379 (PMC12708342; doi:10.1093/pnasnexus/pgaf379)
Supplement: pgaf379_Supplementary_Data [file pgaf379_supplementary_data.pdf]

Supplemental Data

Table S1 Primers used for site-directed mutagenesis

Fragment	Forward Primer (5'-3')	Reverse primer (5'-3')
***SCN5A* gene**	CCAGATCTCTATGGCAATCCA	GAATCTTCACAGCCGCTCTC
**c.715A>G**	GGGCTGAAGACCGTCGTGGGG GCCC	GGGCCCCCACGACGGTCTTCA GCCC
**c.4460T>A**	CCAGGACATCTTCAAGACAGAG GAGCAGA	CCCCCTAACTTTTTCTTCTGTTG GTTGAA

Equation S1 Boltzmann equation for steady-state of activation curve

$$G = \frac{G_{max}}{1 + \exp\ [\frac{(V_{1/2} - V_m)}{k}]}$$

Where G is the conductance, G_{max} is the maximum conductance, V_m is the membrane potential, $V_{1/2}$ is the voltage at which half of the channels are activated and k is the slope factor. Once the curve fitting is completed, extract the optimized values of $V_{1/2}$ and k that indicates the channel's sensitivity to voltage changes.

Equation S2 Boltzmann equation for steady-state of inactivation curve

$$I = \frac{I_{max}}{1 + \exp\ [\frac{(V_{1/2} - V_m)}{k}]}$$

Where I is the peak current amplitude, I_{max} is the maximum peak current amplitude, V_m is the membrane potential, $V_{1/2}$ is the voltage at which half of the channels are inactivated, and k is the slope factor.
